# Supplementary material for: Caspase-1 inhibition alleviates cognitive impairment and neuropathology in an Alzheimer’s disease mouse model
Source: Nat Commun. 2018 Sep 25;9:3916. doi: 10.1038/s41467-018-06449-x (PMC6156230; doi:10.1038/s41467-018-06449-x)
Supplement: Supplementary file 1 — Supplementary Information [file 41467_2018_6449_MOESM1_ESM.pdf]

Caspase-1 inhibition alleviates cognitive impairment, inflammation and amyloid accumulation in an Alzheimer's disease mouse model

Supplementary Figures

Flores et al.,

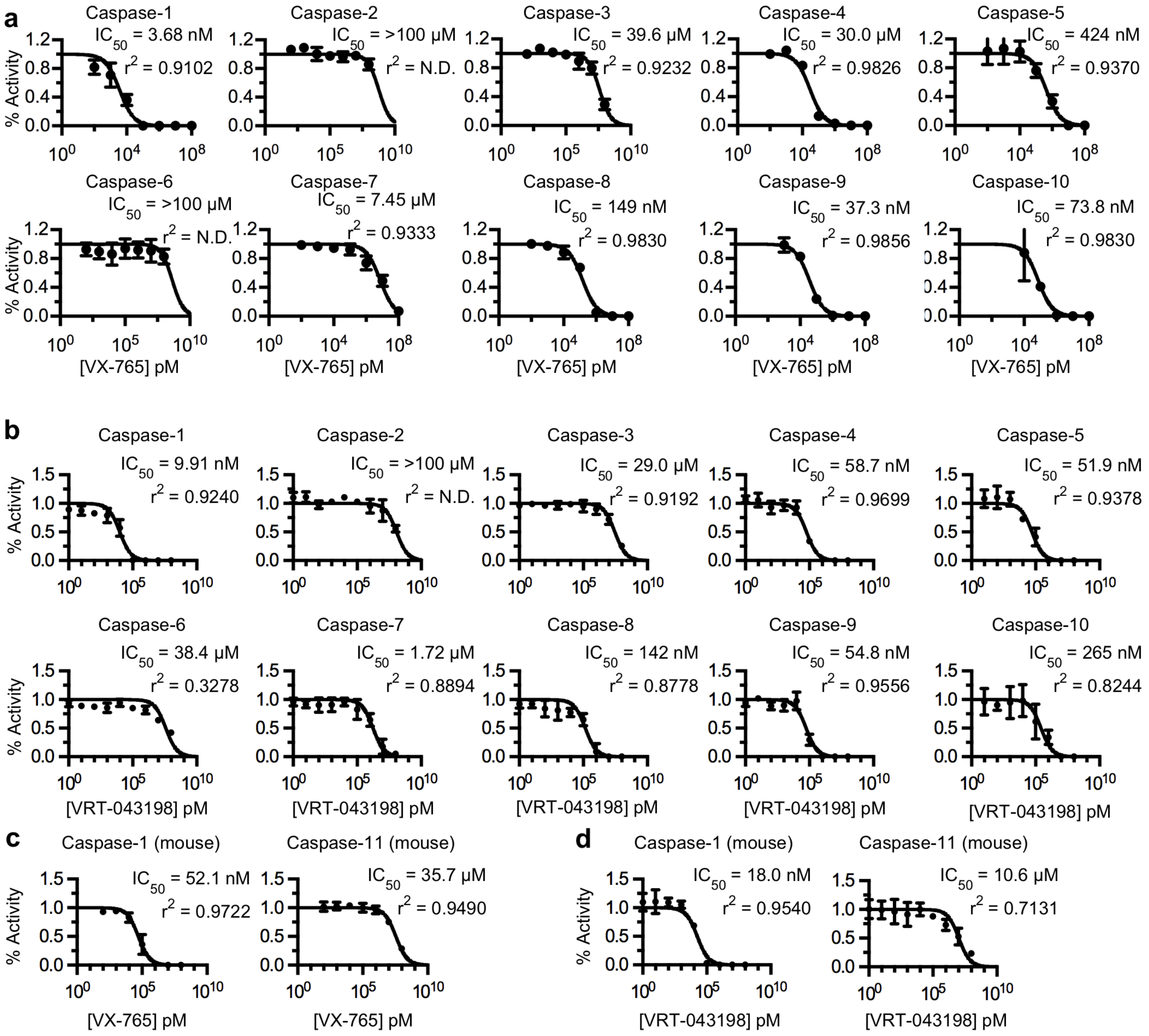

e

VX-765	Brain Cortex (μM)	Brain Hippo (μM)	Plasma (μM)	Ratio (Cortex)	Ratio (Hippo)
WT Mice	4.626	0.877	26.932	**0.172**	**0.033**
	0.378	0.654	14.762	**0.026**	**0.044**
	0.431	11.500	10.839	**0.040**	**1.061**
J20 Mice	1.345	0.709	6.688	**0.201**	**0.106**
	0.648	0.913	8.173	**0.079**	**0.112**
	0.647	1.109	8.790	**0.074**	**0.126**
	0.403	5.700	6.710	**0.060**	**0.849**

VRT-043189	Brain Cortex (μM)	Brain Hippo (μM)	Plasma (μM)	Ratio (Cortex)	Ratio (Hippo)
WT Mice	7.336	2.482	953.900	**0.008**	**0.003**
	1.462	3.373	1202.700	**0.001**	**0.003**
	1.980	20.164	1250.600	**0.002**	**0.016**
J20 Mice	4.041	3.342	720.550	**0.006**	**0.005**
	3.037	3.524	885.060	**0.003**	**0.004**
	2.191	2.564	1199.600	**0.002**	**0.002**
	1.069	10.437	874.660	**0.001**	**0.012**

Supplementary Figure 1. VX-765 and VRT-043198 selectivity and blood brain barrier permeability. (a-b) IC_{50} of **(a)** VX-765 and **(b)** VRT-043198 against human Casp1-10. **(c-d)** IC_{50} of **(c)** VX-765 and **(d)** VRT-043198 against mouse Casp1 and Casp11 (equivalent to human Casp4&5). (**a-d**) Data represents mean of duplicate in vitro experiments and s.e.m. **(e)** Blood brain barrier permeability of VX-765 and VRT-043198 in 3 WT and 4 J20 mice brain cortex, hippocampus and plasma. Ratios of brain levels to plasma levels are calculated.

Supplementary figure 2. Flores et al., Novel treatment against AD

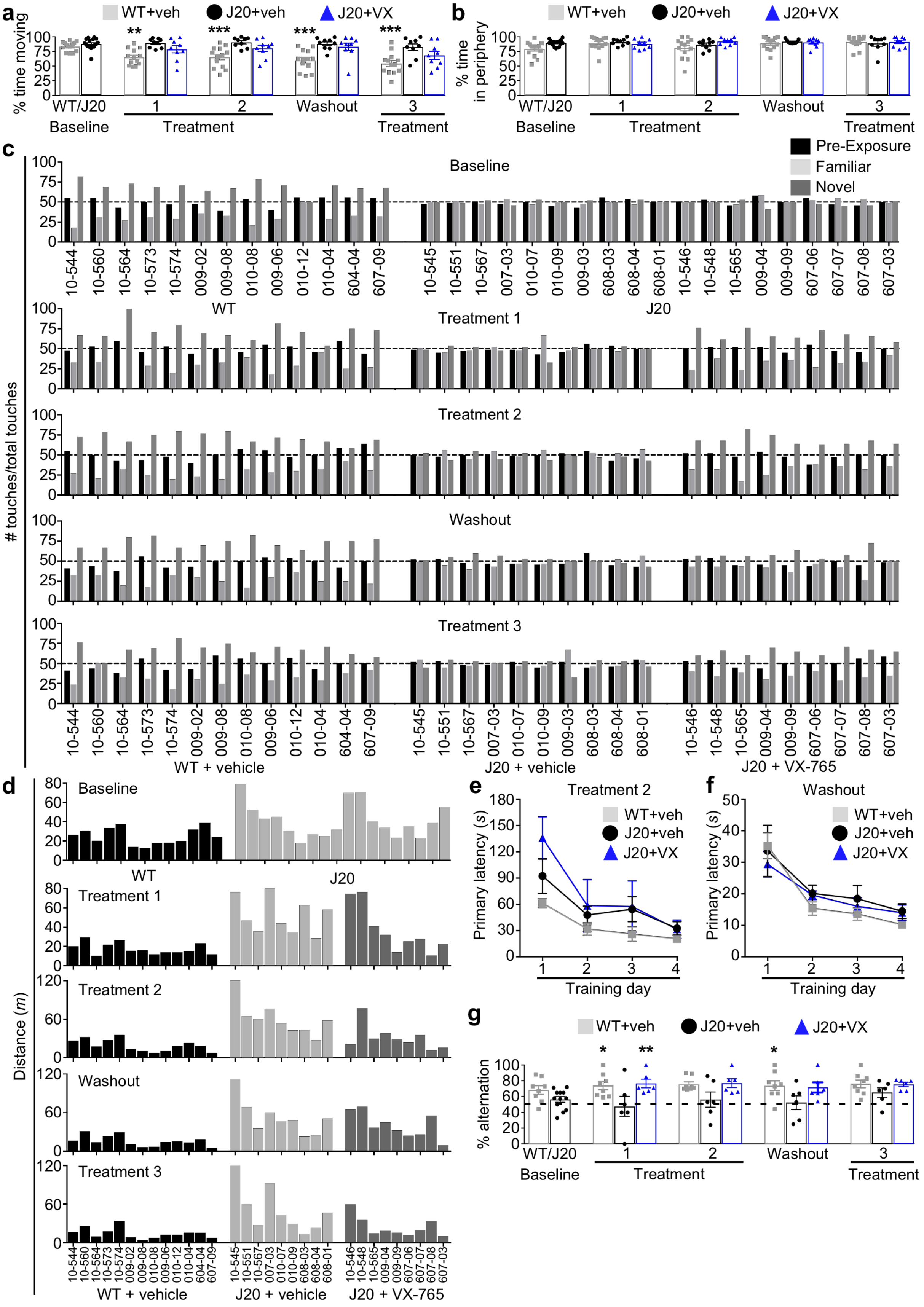

Supplementary Figure 2. Behavioural assessment of vehicle-treated WT and J20 mice and 50 mg kg^{-1} VX-765-treated J20 mice. (a) % time moving [Treatment, $F(2,28) = 10.89$, $p=0.0003$; Time, $F(3,84) = 6.644$, $p=0.0004$, two-way repeated-measures ANOVA, Dunnett's post-hoc versus J20 + vehicle, $^{**}p<0.01$, $^{***}p<0.001$] and **(b)** % time in periphery of vehicle-treated WT (grey squares), vehicle-treated J20 (black circles) and VX-765-treated J20 (blue triangles). Each mouse tested is represented by one symbol. **(c)** NOR # of touches of familiar or novel objects for each individual mouse at each test session. Pre-exposure indicates mice performance with two identical objects, familiar and novel indicates # of touches of familiar and novel objects, respectively. **(d)** Distance travelled in the Open Field task of individual vehicle-treated WT or J20 and VX-765-treated J20. **(e-f)** Barnes maze primary latency to target during learning acquisition at **(e)** T2 and **(f)** WO. **(g)** % alternation in Y-maze apparatus. Two-way repeated measures ANOVA, Dunnett's post-hoc versus J20 + vehicle, $^{*}p<0.05$, $^{**}p<0.01$. Data in a,b,e-g represents mean plus s.e.m.

Supplementary figure 3. Flores et al., Novel treatment against AD

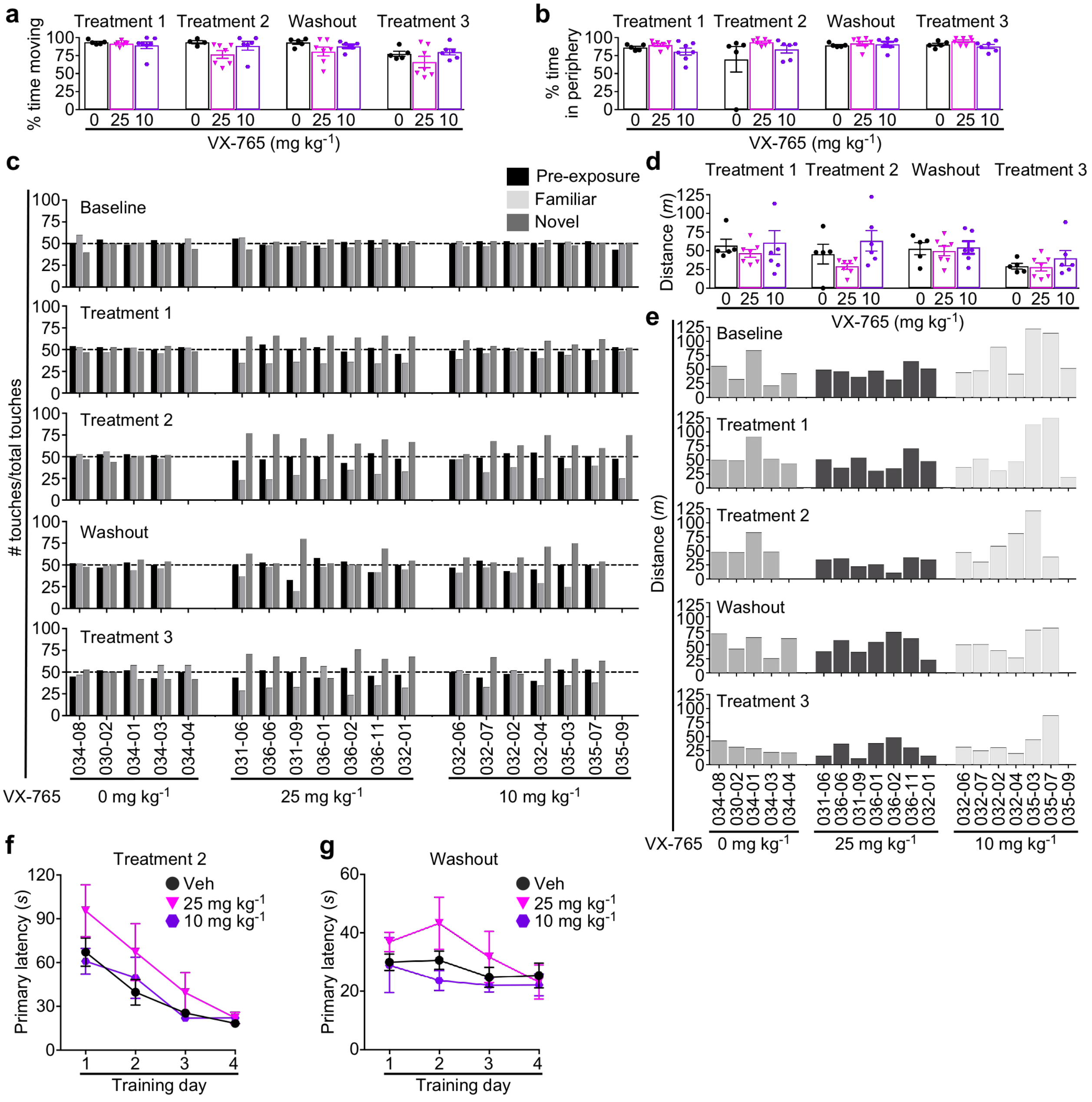

Supplementary Figure 3. Behavioural assessment of vehicle-, or 25 or 10 mg kg^{-1} VX-765-treated J20 mice. (a) % time moving and **(b)** % time in periphery of vehicle-treated J20 (black circles), 25 mg kg^{-1} (pink triangles), or 10 mg kg^{-1} (purple hexagon) VX-765-treated J20 mice. Each mouse tested is represented by one symbol. **(c)** NOR # of touches of familiar and novel object for each individual mouse. **(d)** Distance travelled [Treatment 2, $F(2,14) = 4.106$, $p=0.0395$, ANOVA, Dunnett's post-hoc versus J20 + vehicle (0)] and **(e)** distance travelled by each individual mouse in the Open field task. Two mice (J20 + Vehicle, J20 + 10 mg kg^{-1} VX-765) remained immobile during behavioral testing and were removed from the analysis. **(f-g)** Barnes maze primary latency to target during learning acquisition at **(f)** T2 and **(g)** WO. Data in a,b,d,f-g represents mean and s.e.m.

Supplementary figure 4. Flores et al., Novel treatment against AD

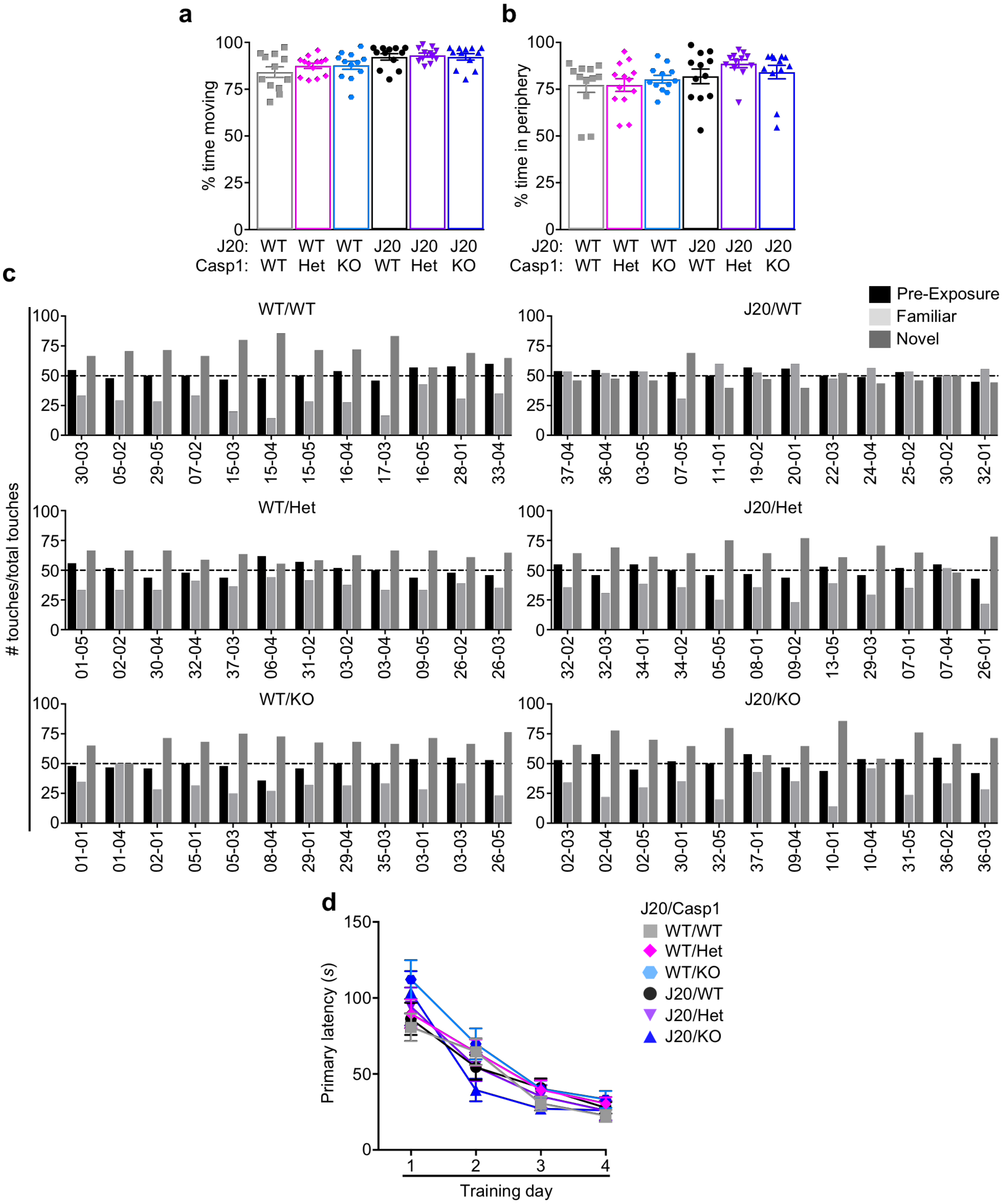

Supplementary Figure 4. Behavioural assessment of J20/KO mice. (a) % time moving and **(b)** % time in periphery of the following genotypes: $J20^{-/-}$/*Casp1*$^{+/+}$ WT/WT (grey squares), $J20^{-/-}$/*Casp1*$^{+/-}$ WT/Het (pink diamond), $J20^{-/-}$/*Casp1*$^{-/-}$ WT/KO (blue hexagons), $J20^{-/+}$/*Casp1*$^{+/+}$ J20/WT (black circles), $J20^{-/+}$/*Casp1*$^{-/+}$ J20/Het (purple triangles), $J20^{-/+}$/*Casp1*$^{-/-}$ J20/KO (blue triangles). Each mouse tested is represented by one symbol. **(c)** NOR # of touches of familiar and novel object for each individual mouse. **(d)** Barnes maze primary latency to target during learning acquisition. Data in a,b.d represents mean and s.e.m.

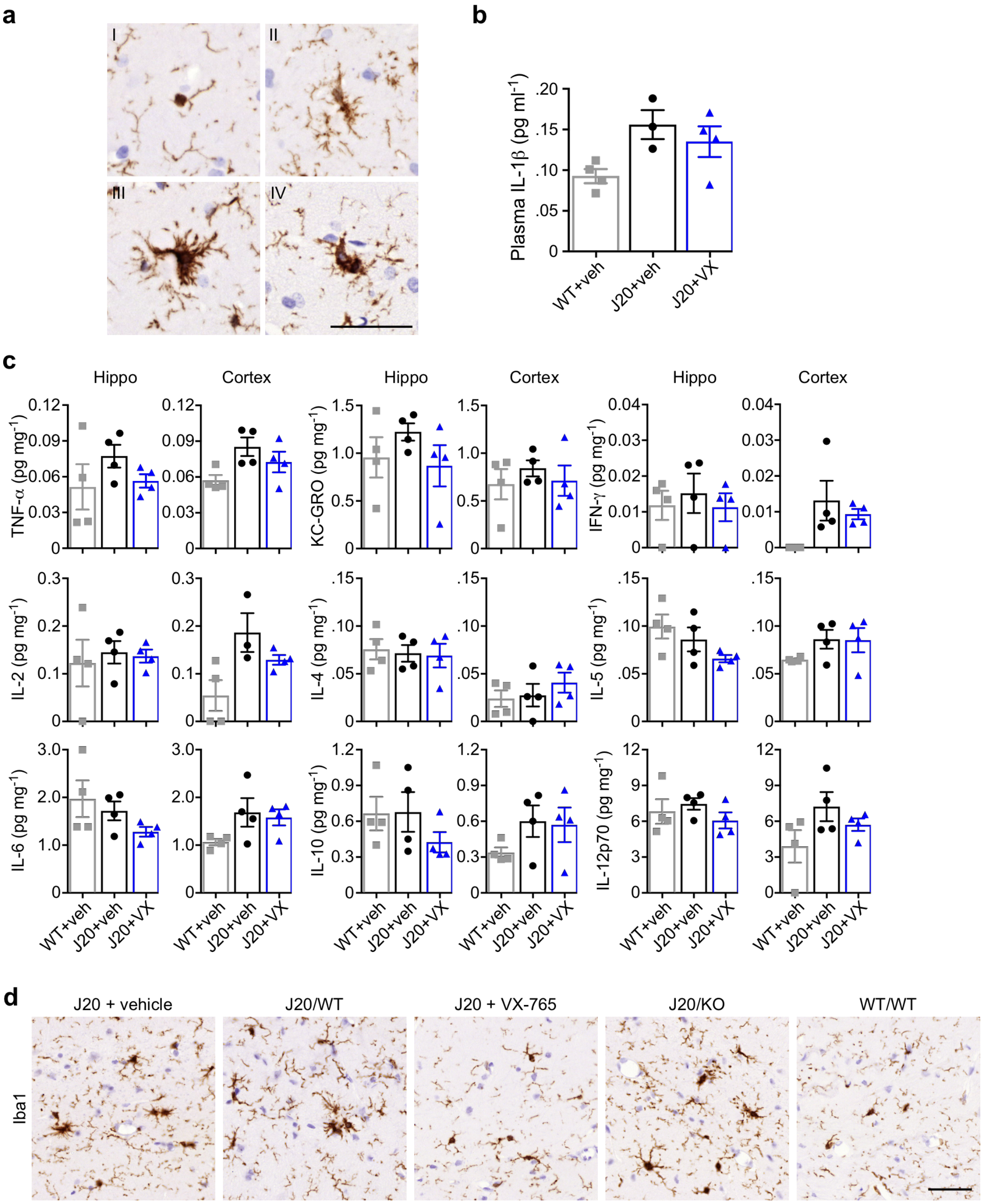

Supplementary Figure 5. Inflammatory marker analyses of vehicle-treated WT and J20, and VX-765-treated J20. (a) Micrographs showing Iba1 I, II, III, IV microglial subtypes. **(b)** IL-1β plasma levels after T3 in periphery of vehicle-treated WT (grey squares), vehicle-treated J20 (black circles) and VX-765-treated J20 (blue triangles). **(c)** Cytokine levels in vehicle-treated WT and J20 and in VX-765-treated J20 brain hippocampi and cortex (n=4/group). **(d)** Iba1 immunopositive staining comparing 8-month old vehicle-treated J20, J20/WT, VX-765-treated J20, J20/*Casp1*$^{-/-}$ (J20/KO), and WT/WT mice hippocampi. Each mouse tested is represented by one symbol. Data represents mean and s.e.m. Scale bar in **(a)** = 20 μm, **(d)** = 50 μm.

Supplementary figure 6. Flores et al., Novel treatment against AD

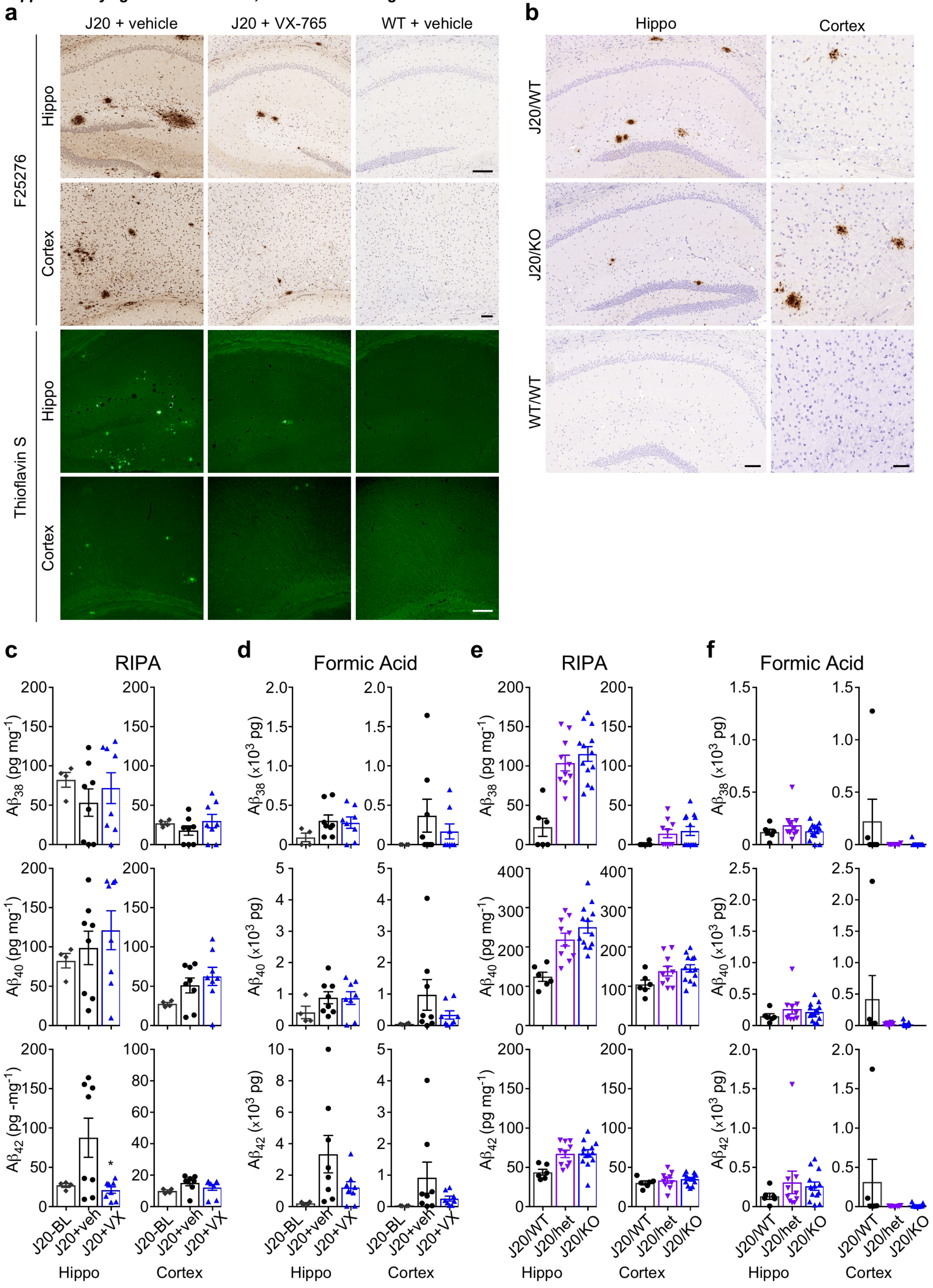

Supplementary Figure 6: Aβ stained with Thioflavin S or anti-A$\beta_{1\text{-}40}$ F25276 antiserum in mice hippocampi or cortex. (a) Comparison of anti-Aβ F25276 immunostaining and thioflavin S-stained in 8-month old vehicle-treated WT and J20 and in VX-765-treated J20 brain hippocampi and cortex (after T3). **(b)** Anti-Aβ immunopositive staining in 8-month-old J20/WT, J20/*Casp1*$^{-/-}$ (J20/KO), and WT/WT mice. **(c)** RIPA-soluble **(d)** and formic acid soluble Aβ_{38}, Aβ_{40}, and Aβ_{42} levels in baseline J20 (dark grey diamonds), vehicle-treated J20 (black circles) and VX-765-treated J20 (blue triangles) brain hippocampi and cortex measured by ELISA [Aβ_{42} RIPA-Hippo, $F(2,17) = 4.795$, $p=0.0223$]. **(e)** RIPA-soluble **(f)** and formic acid soluble Aβ_{38}, Aβ_{40}, and Aβ_{42} levels in J20$^{-/+}$/*Casp1*$^{+/+}$ J20/WT (black circles), J20$^{-/+}$/*Casp1*$^{-/+}$ J20/Het (purple triangles), and J20$^{-/+}$/*Casp1*$^{-/-}$ J20/KO (blue triangles). Each mouse tested is represented by one symbol. Data represents mean and s.e.m. **(c-f)** ANOVA, Dunnett's post-hoc versus J20 + vehicle **(c-d)** or J20/WT **(e-f)**, *$p<0.05$. Scale bars = 50 μm.

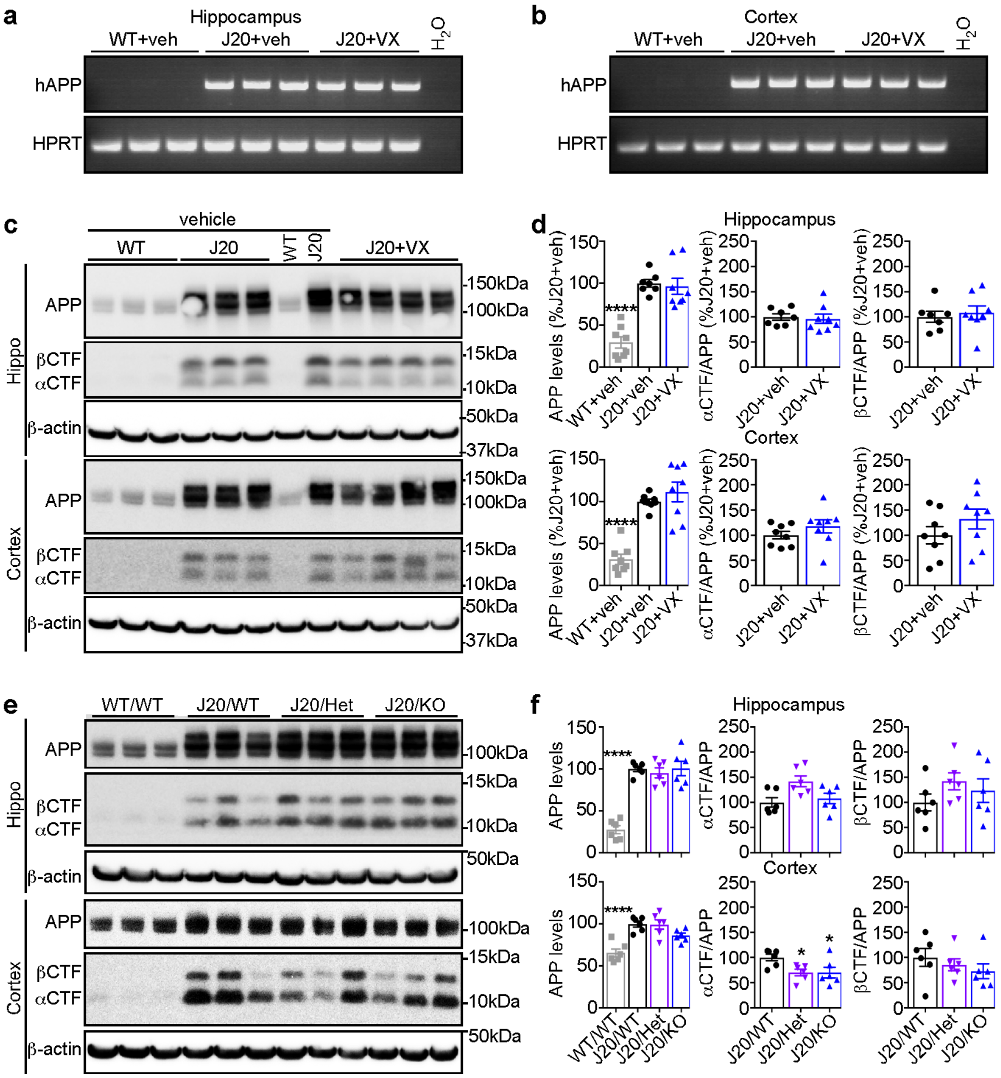

Supplementary Figure 7: APP and CTFs levels after VX-765 or *Casp1* KO. (a-b) Ethidium bromide-stained agarose gel of human APP and HPRT RT-PCR amplicons from the **(a)** hippocampus and (**b**) cortex of vehicle-treated WT and J20, and VX-765-treated J20. **(c-d)** APP and CTF (anti-APP C-terminal antibody) western blots, and quantitative analyses in the brain hippocampi and cortex of vehicle-treated WT (grey squares), vehicle-treated J20 (black circles) and VX-765-treated J20 (blue triangles) [APP hippo, $F(2,20) = 28.81$, $p<0.0001$; APP cortex, $F(2,20) = 31.2$, $p<0.0001$]. Quantitative results represented as ratios of APP levels over β-actin or CTFs over APP and expressed as percentage of J20 + vehicle. ANOVA, Dunnett's post-hoc versus J20 + vehicle, ****$p<0.0001$. **(e-f)** APP and CTF western blots, and quantitative analyses in the brain hippocampi and cortex of $J20^{-/-}$/$Casp1^{+/+}$ WT/WT (grey squares), $J20^{-/+}$/$Casp1^{+/+}$ J20/WT (black circles), $J20^{-/+}$/$Casp1^{-/+}$ J20/Het (purple triangles), and $J20^{-/+}$/$Casp1^{-/-}$ J20/KO (blue triangles) [APP hippo, $F(3,20) = 26.04$, $p<0.0001$; APP cortex, $F(3,20) = 14.07$, $p<0.0001$; αCTF cortex, $F(2,15) = 4.45$, $p= 0.0304$]. Quantitative results represented as ratios of APP levels over β-actin or CTFs over APP and expressed as percentage ± SEM of J20/WT. ANOVA, Dunnett's post-hoc versus J20/WT, *$p<0.05$, ****$p<0.0001$. Each mouse tested is represented by one symbol. Data represents mean and s.e.m.

Supplementary Figure 8. Flores et al., Novel treatment against AD.

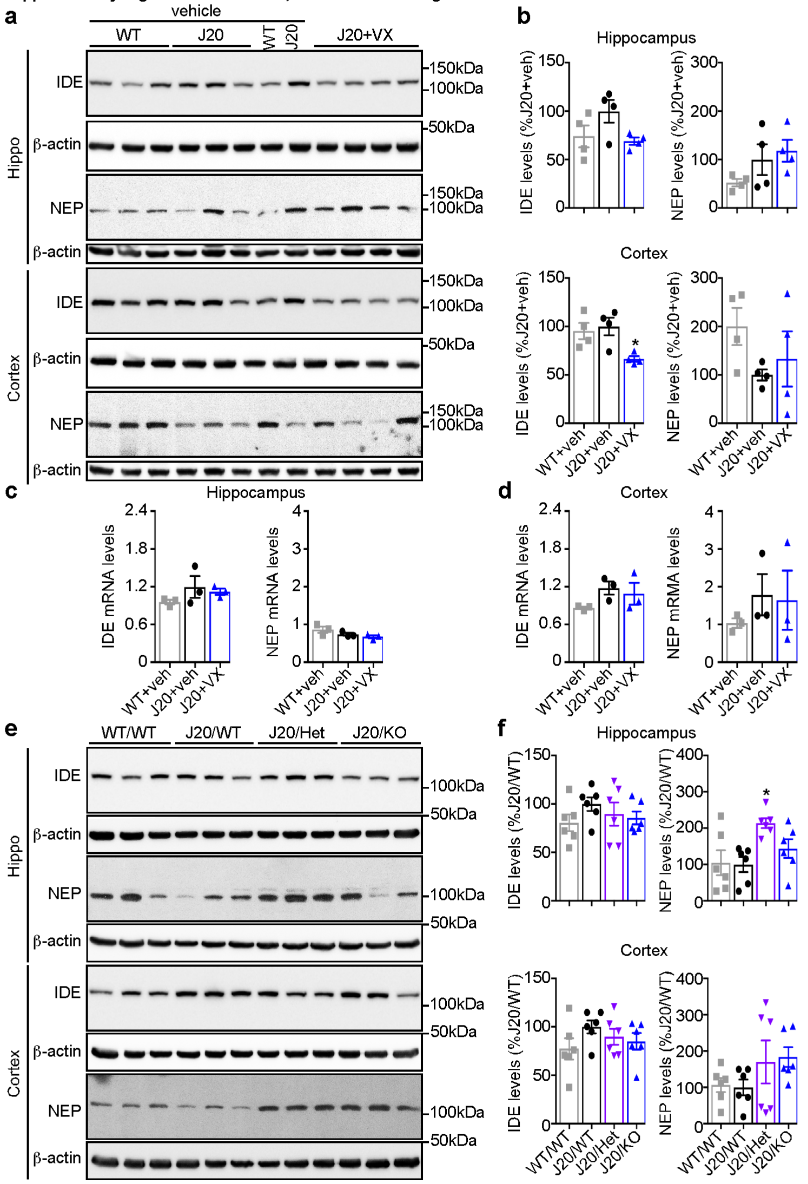

Supplementary Figure 8: IDE and NEP levels after VX-765 or *Casp1* KO. (a-b) IDE and NEP western blots, and quantitative analyses in the brain hippocampi and cortex of vehicle-treated WT (grey squares), vehicle-treated J20 (black circles) and VX-765-treated J20 (blue triangles) [IDE cortex, $F(2,9) = 6.098$, $p=0.0212$]. Quantitative results represented as ratios of IDE or NEP levels over β-actin and expressed as percentage of J20 + vehicle. ANOVA, Dunnett's post-hoc versus J20 + vehicle, *$p<0.05$. **(c-d)** mRNA levels of IDE and NEP in the **(c)** hippocampus and **(d)** cortex of vehicle-treated WT and J20, and VX-765-treated J20. **(e-f)** IDE and NEP western blots, and quantitative analyses in the brain hippocampi and cortex of $J20^{-/-}$/*Casp1*$^{+/+}$ WT/WT (grey squares), $J20^{-/+}$/*Casp1*$^{+/+}$ J20/WT (black circles), $J20^{-/+}$/*Casp1*$^{-/+}$ J20/Het (purple triangles), and $J20^{-/+}$/*Casp1*$^{-/-}$ J20/KO (blue triangles) [NEP Hippo, $F(3,20) = 4.537$, $p=0.0139$]. Quantitative results represented as ratios of IDE or NEP levels over β-actin and expressed as percentage of J20/WT. ANOVA, Dunnett's post-hoc versus J20/WT, *$p<0.05$. Each mouse tested is represented by one symbol. Data represents mean and s.e.m.

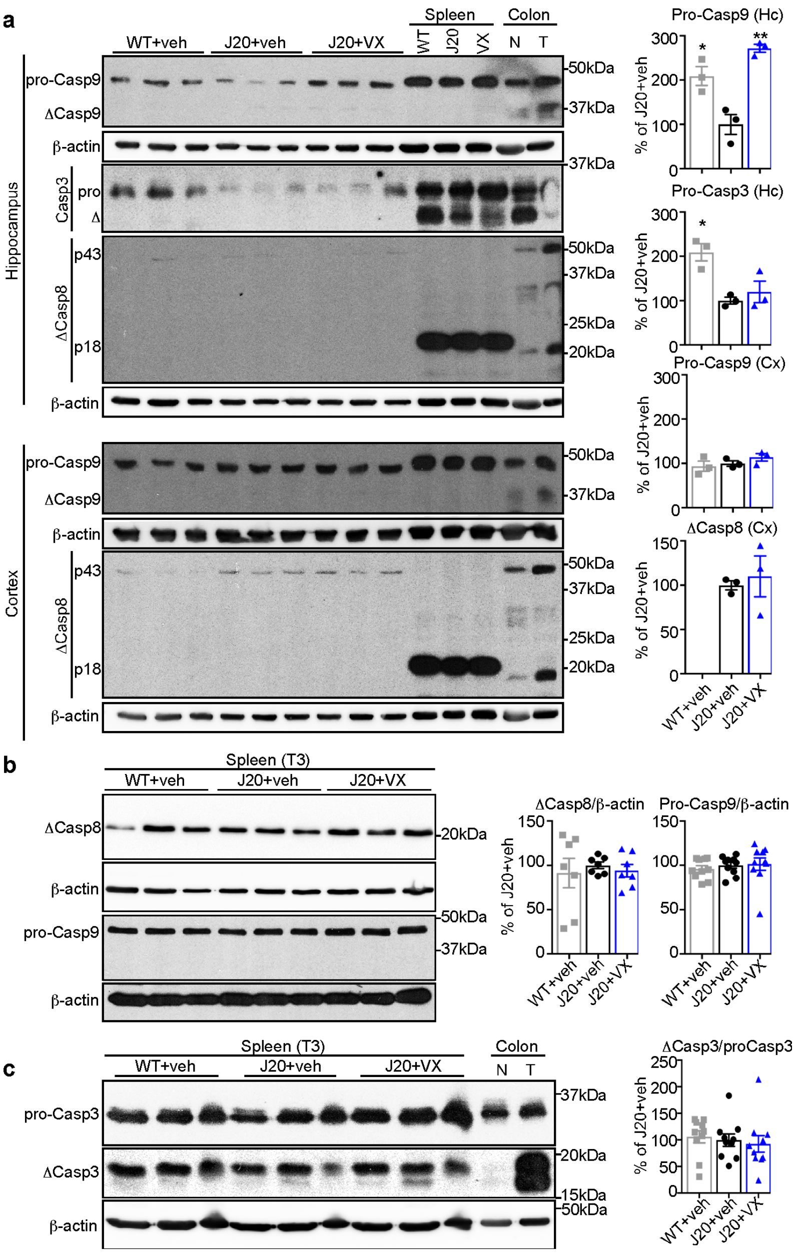

Supplementary Figure 9. Caspase-3, -8 and -9 activation after VX-765. (a) Western blot and quantitative analyses of Casp3, Casp8, and Casp9 expression in the hippocampus and cortex of vehicle-treated WT (grey squares), vehicle-treated J20 (black circles) and VX-765-treated J20 (blue triangles). Mouse spleen and colon tissues were used as controls. N: normal, T: tumor. **(b-c)** Representative **(b)** cleaved Casp8 (ΔCasp8) and pro-Casp9, and **(c)** Casp3 western blot and quantitative analyses in the spleen of vehicle-treated WT, J20, and VX-765-treated J20 mice. Quantitative results represented as ratios of **(a,b)** pro-Casp3, ΔCasp8 or pro-Casp9 over β-actin, or **(c)** cleaved Casp3 (ΔCasp3) over pro-Casp3 and are expressed as percentage of J20 + vehicle. ANOVA, Dunnett's post-hoc versus J20 + vehicle, $*p<0.05$, $**p<0.01$. Each mouse tested is represented by one symbol. Data represents mean and s.e.m.

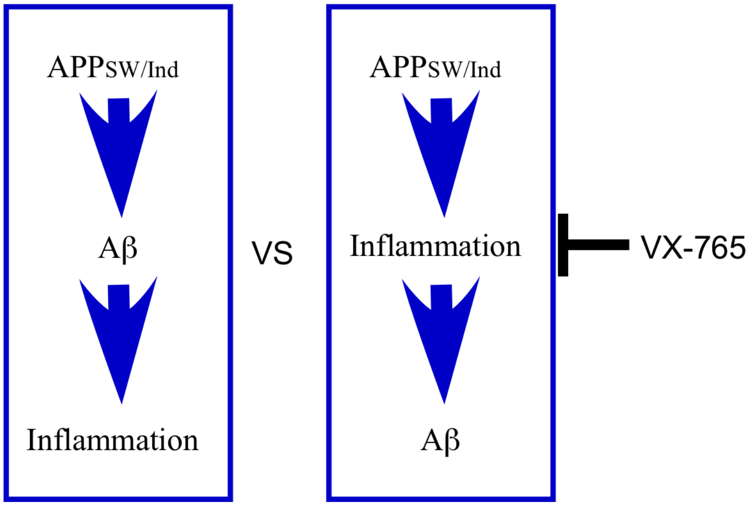

Supplementary Figure 10: Schematic diagram to illustrate pathway inhibited by VX-765. The $APP^{Sw/Ind}$ transgene is predicted to increase Aβ levels, which then generate inflammation through microglial activation (Left panel). Our results are more consistent with a model where the $APP^{Sw/Ind}$ transgene first induces inflammation, possibly in neurons, and then leads to increased Aβ (Right panel). By inhibiting Casp1, VX-765 blocks inflammation and subsequent Aβ accumulation.

Supplementary figure 11. Flores et al., Novel treatment against AD

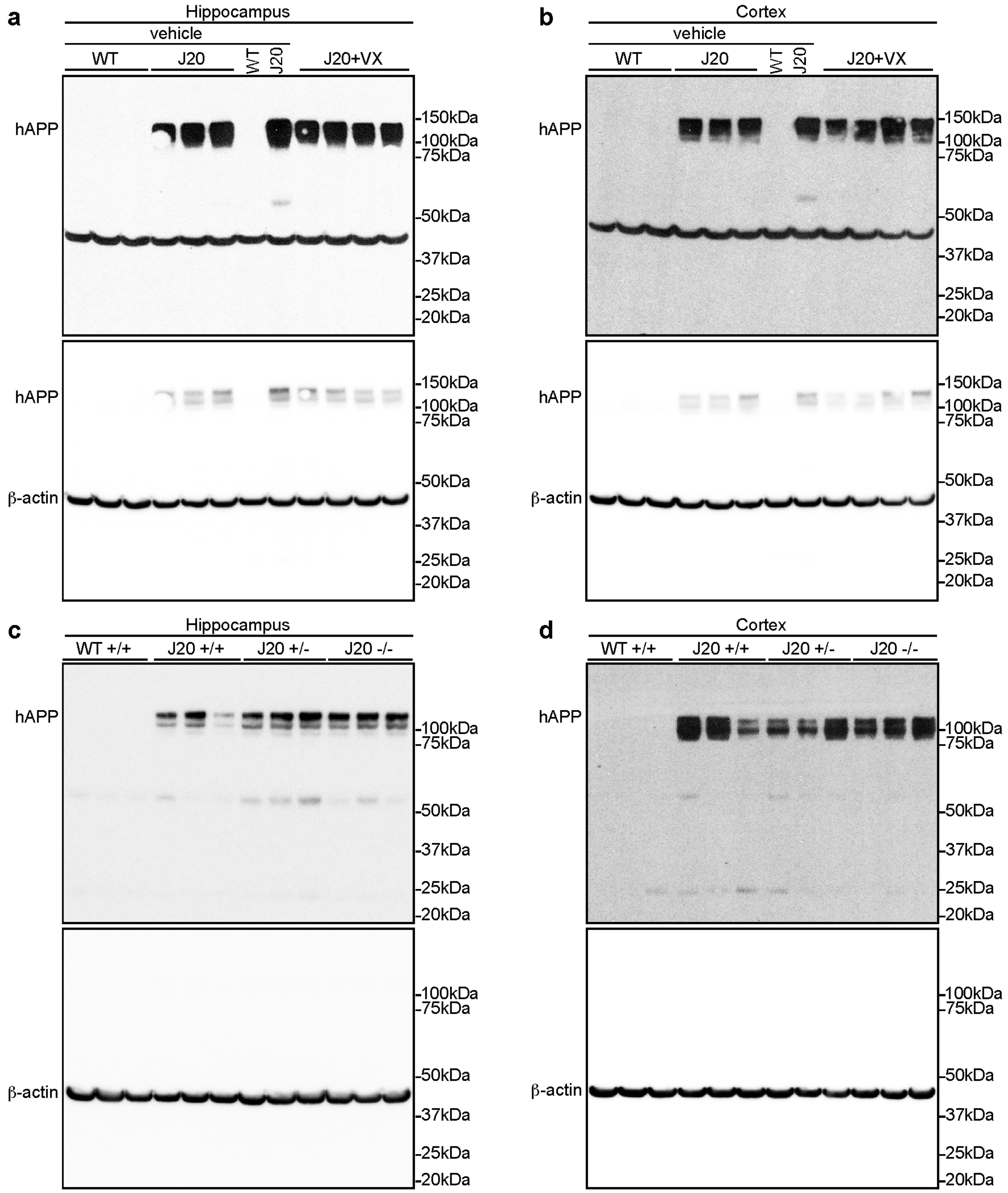

Supplementary Figure 11: VX-765 prevents progressive Aβ accumulation in J20 mice. (a-b) Full western blot images of hAPP and β-actin in the **(a)** hippocampus and **(b)** cortex of vehicle-treated WT, vehicle-treated J20 and VX-765-treated J20 from Fig. 5l. **(c-d)** Full western blot images of hAPP and β-actin in the **(c)** hippocampus and **(d)** cortex of cortex of J20$^{-/-}$/*Casp1*$^{+/+}$ WT/WT, J20$^{-/+}$/*Casp1*$^{+/+}$ J20/WT, J20$^{-/+}$/*Casp1*$^{-/+}$ J20/Het, and J20$^{-/+}$/*Casp1*$^{-/-}$ J20/KO from Fig. 5n.
